# Mild Traumatic Brain Injury in Older Adults: Are Routine Second cCT Scans Necessary?

**DOI:** 10.3390/jcm10173794

**Published:** 2021-08-25

**Authors:** Valeska Hofmann, Christian Deininger, Stefan Döbele, Christian Konrads, Florian Wichlas

**Affiliations:** 1 BG Trauma Centre, Department of Trauma and Reconstructive Surgery, University of Tübingen, 72076 Tübingen, Germany; sdoebele@bgu-tuebingen.de (S.D.); ckonrads@bgu-tuebingen.de (C.K.); 2 Department of Orthopedics and Traumatology, University Hospital Salzburg, 5020 Salzburg, Austria; christian.deininger@hotmail.com (C.D.); fwichlas@icloud.com (F.W.)

**Keywords:** anticoagulation, concussion, geriatric trauma, overdiagnosis

## Abstract

Fall-related hospitalizations among older adults have been increasing in recent decades. One of the most common reasons for this is minimal or mild traumatic brain injury (mTBI) in older individuals taking anticoagulant medication. In this study, we analyzed all inpatient stays from January 2017 to December 2019 of patients aged > 75 years with a mTBI on anticoagulant therapy who received at least two cranial computer tomography (cCT) scans. Of 1477 inpatient stays, 39 had primary cranial bleeding, and in 1438 the results of initial scans were negative for cranial bleeding. Of these 1438 cases, 6 suffered secondary bleeding from the control cCT scan. There was no significance for bleeding related to the type of anticoagulation. We conclude that geriatric patients under anticoagulant medication don’t need a second cCT scan if the primary cCT was negative for intracranial bleeding and the patient shows no clinical signs of bleeding. These patients can be dismissed but require an evaluation for need of home care or protective measures to prevent recurrent falls. The type of anticoagulant medication does not affect the risk of bleeding.

## 1. Introduction

Older populations are growing continuously in high income countries (HIC) [1]. In addition to the increasing number of comorbidities and medications, musculoskeletal decay has become relevant for this part among the population [2]. Osteopenia, sarcopenia, and dementia impede musculoskeletal coordination and lead to recurrent falls of geriatric patients [3,4,5]. As a result of the aging process, these patients have difficulty performing daily tasks; a fall represents an early symptom of coping-failure [6]. These patients are likely to require care by family or nursing professionals sooner or later. A fall is the main reason for orthopaedic-traumatological admissions for older patients [7,8]. Frequent reasons for hospital admission include fractures of the proximal femur, the spine, the proximal humerus, and the distal radius [9]. Besides these fractures, traumatic brain injury (TBI) caused by a fall is an increasing reason for hospitalization in older patients [10]. In older patients, a fall is the main reason for a traumatic brain injury (51%), followed by car accidents (9%) [11].

The first goal in the treatment of TBI in older adults is to diagnose and exclude intracranial bleeding. A cranial computer tomography (cCT) scan is the imaging technique of choice for the diagnosis of intracranial bleeding. Guidelines for its indication are well established and validated [12,13]. Patient’s age greater than 65 has been recognized as being a factor towards the indication for a cCT.

As comorbidities and medications for geriatric patients increase, the use of oral anticoagulants and antiplatelets (ACAP) has increased significantly [14]. The risk for bleeding complications after trauma for patients under ACAP-treatment has been widely described [15,16]. Furthermore, the use of ACAP has also been implemented as a risk factor in the guidelines for primary cCT for TBI. Most clinical departments have already established a protocol for diagnosing delayed secondary bleeding, but no guidelines for the detection of such intracranial bleeding has been described.

The aim of this study was to investigate the risk for a delayed intracranial bleeding in older patients taking ACAP after a fall with minimal or mild TBI (mTBI). The definition of minimal or mild TBI was based on the GCS interval of the Head Injury Severity Score (HISS) [17]. We evaluated the incidence of a delayed intracranial bleeding after primary negative cCT of older patients admitted to the hospital. Additionally, we determined the rate of readmission to the hospital, the duration of hospital stay, the ACAP taken, and the primary bleeding.

The incidence of the delayed intracranial bleeding should determine the need for a second cCT control and for a hospital stay. The number of readmissions and the duration of the hospital stay should show the necessity for caregiving. The ACAP taken and the number of primary bleeding should estimate the risk for intracranial bleeding.

## 2. Materials and Methods

We retrospectively analyzed data from 1477 inpatient stays of 1129 patients with mTBI admitted to a level-one-trauma center of a university hospital from January 2017 to December 2019, in Salzburg, Austria.

Inclusion criteria were:

- mTBI.
- Age > 75 years.
- Taking ACAP at time of injury.
- Low impact trauma mechanism by fall.
- Two or more cCT scans.

Exclusion criteria were:

- Other concomitant injuries that would indicate inpatient treatment.
- High impact trauma.
- One cCT Scan only.

Demographic data of the study population are shown in Table 1.

On the 1477 inpatient stays, 3021 cCT scans were performed; in 1437 cases 2 cCT scans, in 27 cases 3, in 7 cases 4, in 2 cases 5, in 2 cases 6, in 1 case 7, and in 1 case 9 cCTs were conducted. The first cCT scan was conducted on the day of admission and the second 24 h later.

The CT scanner was a 16-slice scanner (Siemens Somatom Emotion 16, Siemens, Erlangen/Germany). The scans were evaluated by an on-call radiological and trauma consultant and the authors of the study. Scans that could not be diagnosed sufficiently due to artifacts were excluded.

Primary negative cCT scans for an intracranial bleed were recorded if they were positive on the control scan. When the cCT control scan was positive for intracranial bleeding, we evaluated the bleeding on the cCT control scans and the resulting hospital stay.

The number of readmission and the duration of the hospital stay of all patients was evaluated. The intake of antiplatelet or anticoagulant medication was noted and possible association of such a medication with the incidence of primary and secondary bleeding was analyzed.

## 3. Results

### 3.1. Readmission and Duration of Stay

Inpatient admission occurred in 1129 patients; in 931 (83%) patients once; and in 198 (17%) multiple times. This resulted in 1477 admissions in total (Table 2).

The duration of inpatient stays ranged from 1 to 37 days. The regular stay was 2 days in 1271 cases. Of the remaining, 25 patients could not be mobilized and dismissed at home, 21 had elevated infection parameters and were treated anti-infectively, and 12 needed more than traumatological consultation. For the rest, the reason for prolonged hospitalization could not be determined retrospectively.

### 3.2. cCT Control Scans, Hospital Stay

Of 1477 inpatient stays, 39 (2.64%) cases had a primary bleeding and 1438 (97.36%) had an initial negative scan. Secondary intracranial bleeding was present in 6 of these 1438 cases, as shown in Table 3.

In the cases with an initial negative scan and no secondary bleeding (*n* = 1432), a total of 2852 cCT scans were performed. In these cases, the mean stay was 1.7 days.

In the six patients with a secondary bleeding, two had one cCT control, three had two, and one had three after the initial cCT scan. The intracranial bleeding was an intraparenchymal hemorrhage in four cases, a subdural hematoma in one case, and a combination of both in one case. All six were treated conservatively and five were dismissed home without any further therapy. They had no symptoms or their bleeding was decreasing in the cCT control. The patient with the combined intraparenchymal hemorrhage and subdural hematoma had symptoms with an increasing unconsciousness. He had increasing bleeding on cCT scans and died seven days after admission at the age of eighty-nine. The mean hospital stay of these six patients was four days.

In the cases with a primary bleeding (*n* = 39), 134 cCT scans were performed. Most patients received three cCT scans, and one of them nine scans (Table 4). In these cases, the mean in-hospital stay was 6.3 days.

### 3.3. Oral Anticoagulants and Antiplatelet Medication

Of 1477 cases, 1443 patients were taking one ACAP, 33 were taking a dual medication, and 1 patient was taking a triple combination. Every double medication was a combination of an oral anticoagulants with an antiplatelet agent.

Every patient with a primary (39) or secondary (6) bleeding has been taking one ACAP. In the cases where bleeding did not occur (1398), they were taking an ACAP. Table 5 shows the intake of one ACAP in every case.

Two patients with a secondary intracranial bleeding had been taking acetylsalicylic acid, two clopidogrel, one apixaban, and one acenocoumarol. The comparison of patients with a secondary bleeding and without intracranial bleeding regarding the intake of antiplatelet (4/720) or anticoagulant medication (2/678) was statistically not significant (Chi-square statistic is 0.5548, *p* = 0.456367; not significant at *p* > 0.05).

In 39 cases with a primary bleeding, the difference between antiplatelet and anticoagulant intake (Table 6) was also statistically not significant in comparison with the cases without bleeding (Chi-square statistic is 1.9976, *p* = 0.157549; not significant at *p* > 0.05).

Two patients were treated surgically by craniotomy, one of them died after 8 days. Another patient died without any surgical therapy. The other patients were treated conservatively and left the hospital after the bleeding subsided.

## 4. Discussion

Of 1477 admitted cases aged above 75 years and taking ACAP, 39 had a primary intracranial bleeding and only 6 developed a delayed intracranial bleeding after 24 h. Although guidelines for cCT scans exist, every patient with anamnestic fall resulting in a mTBI and anticoagulating medication received a cCT scan. The Canadian head CT rule explicitly excludes bleeding disorders but indicates a cCT scan for minor head injury for patients older than 64 years [18]. The New Orleans Criteria indicate the cCT scan for patients who are older than 59 years with minor head trauma and loss of consciousness and are neurologically normal [19]. They do not consider bleeding disorders. The Scandinavian guidelines consider patients age > 64 and coagulation disorders [13]. They differentiate between patients > 64 years taking antiplatelet and patients of any age taking a therapeutic anticoagulation. The first group is recommended to undergo a cCT scan, or 12 h observation, and discharge if the cCT scan is negative. The second group is recommended to undergo a cCT scan and admission > 24 h independent of the findings. In our collective, the difference of these bleeding disorders was not significant (although the number of patients with intracranial bleeding was low). The number of patients taking antiplatelet and those taking other anticoagulant medication were almost the same. Nevertheless, we couldn’t find any statistical significance.

The patients of this study fulfill the guidelines for a primary cCT scan according to the Scandinavian guidelines [13]. Although no guidelines for cCT scan control after mTBI exist, each patient received a cCT control 24 h after the initial scan. A meta-analysis of studies identified 0.60% of secondary intracranial bleeding and 0.13% of neurosurgical intervention for patients (*n* = 1494) with mild traumatic brain injury, negative primary cCT scan, and anticoagulation using vitamin K antagonists [20]. The authors of this study therefore do not recommend cCT controls as part of a standard procedure. The patients of this study showed 0.42% risk for secondary intracranial bleeding and none for neurosurgical intervention. We demonstrated that for these patients the cCT control had no therapeutic consequence besides the longer hospital stay (4 days). The need for a cCT control is at least to be doubted, especially when the consequence is two additional days of hospital stay. The second cCT scan means that the criteria for over-diagnosis have been attained [21]. Based on the data of this study, we recommend that if the primary cCT scan is negative for a traumatic bleeding and the patient has no clinical symptoms, a possible cCT control in an outpatient setting would be sufficient. Even the outpatient control can be discussed since it has no therapeutic consequence but it takes an effort for the older patient. Geriatric patients do not tolerate a change in their surroundings or an interruption in their daily routine well [22].

The primary intracranial bleeding in the study population taking ACAP was 2.64%. The role of cCT controls in these patients is to detect the development of bleeding in order to make decisions regarding further therapy. Guidelines for surgery exist and they mainly consist in craniotomy [23].

In the study population, more than 60% of patients with primary bleeding have been taking antiplatelet medication. Contrary to the Scandinavian guidelines, we could not find a difference in bleeding risk between antiplatelet medication and anticoagulation. We did not find a higher risk of intracerebral bleeding for patients on anticoagulation.

The admission of older patients in traumatological-orthopedic departments has been increasing dramatically during the last decade [24]. Mild traumatic brain injury is one of the most common reasons for emergency department admission and falls are often related to medical conditions (e.g., syncope) [25]. In our study cohort, 17% of the patients were admitted more than once for the same reason (mTBI after a fall). In our opinion, patients represent a lack of preventive measures for falls among geriatrics and care of the older adults. Although it is well known that a fall in older adults is a major reason for hospitalization, 17% of the patients in our study cohort were not taken care of after the first fall. A fall in the older patient should always alert every treating discipline as it probably is a sign for physiologic deterioration and the loss of the ability to handle daily tasks. This seems to be underlined by another fact that in 25 cases of 1432 (1.7%) without bleeding, the patients cannot be dismissed back home because they cannot be mobilized adequately. The fall of these patients seems to be a final surrender for living alone. Most falls in older adults have only minor injury consequences. However, the resulting pain and discomfort often leads to a loss of self-confidence and independence [26]. These patients can’t be returned injudiciously to their homes without questioning the surrounding care. In another 21 cases (1.4%), patients couldn’t be dismissed after mTBI since they had elevated infection parameters. This could be the result of a rapid deterioration of health of the older adults due to infection. Secondarily, this can lead to a faster reduction of independence at home. However, we believe that a fall for an older person is more than just an accident; it marks the initiation of inability to deal with daily living activities.

The main limitation of this study is its retrospective design. Although it was a single-institution study we were able to include a large sample. It is comparable to existing systematic reviews on this topic.

## 5. Conclusions

In conclusion, the results of this study suggest that after initial cCT in geriatric patients without primary intracranial bleeding under anticoagulant medication, a secondary control cCT is not necessary if no clinical signs of intracranial bleeding are apparent. For patients without relevant concomitant injuries requiring inpatient treatment, the inpatient stay can be shortened or outpatient treatment can be provided. In geriatric patients, the focus should be on home care and fall prevention.

## Figures and Tables

**Table 1:** Patient demographics.

Parameter	Males	Females	∑	Ø
*N*	440	689	1129	
Age				85.56 (75–105)

**Table 2:** Number of hospital admissions of geriatric patients with mTBI due to a fall.

Hospital Admissions	Patients (*n*)
1	931
2	123
3	42
4	16
5	8
6	4
7	0
8	1
9	2
10	2

**Table 3:** Distribution of cCT findings with “bleeding” and “no bleeding” among all patients.

cCT	Bleeding	No Bleeding	Total Number
Primary	39 (2.64%)	1438 (97.36%)	1477 (100%)
Secondary	6 (0.42%)	1432 (99.58%)	1438 (100%)

**Table 4:** Number of cCT-scans of patients with primary bleeding.

Number of cCT-Scans	Cases (*n*)
2	9
3	18
4	6
5	2
6	2
7	1
8	0
9	1

**Table 5:** ACAPs taken by patients admitted to in-hospital stay due to mTBI.

	*n*	Percent
Acetylsalicylic Acid (100 mg)	661	46%
Clopidogrel	88	6%
Apixaban	192	13%
Dabigatran	95	7%
Rivaroxaban	206	14%
Phenprocoumon	74	5%
Acenocoumarol	111	8%
Others	16	1%
	1443	100.00

**Table 6:** ACAP of patients with primary bleeding.

	*n*	Percent
Acetylsalicylic Acid (100 mg)	21	54%
Clopidogrel	4	10%
Apixaban	4	10%
Dabigatran	2	5%
Rivaroxaban	1	3%
Phenprocoumon	2	5%
Acenocoumarol	5	13%
Others	0	0%
	39	100.00%

## Data Availability

The data presented in this study are available on request from the corresponding author. The data are not publicly available due to privacy.
